# Supplementary figures and images for: Fully automated sequence alignment methods are comparable to, and much faster than, traditional methods in large data sets: an example with hepatitis B virus
Source: PeerJ. 2019 Jan 3;7:e6142. doi: 10.7717/peerj.6142 (PMC6321758; doi:10.7717/peerj.6142)

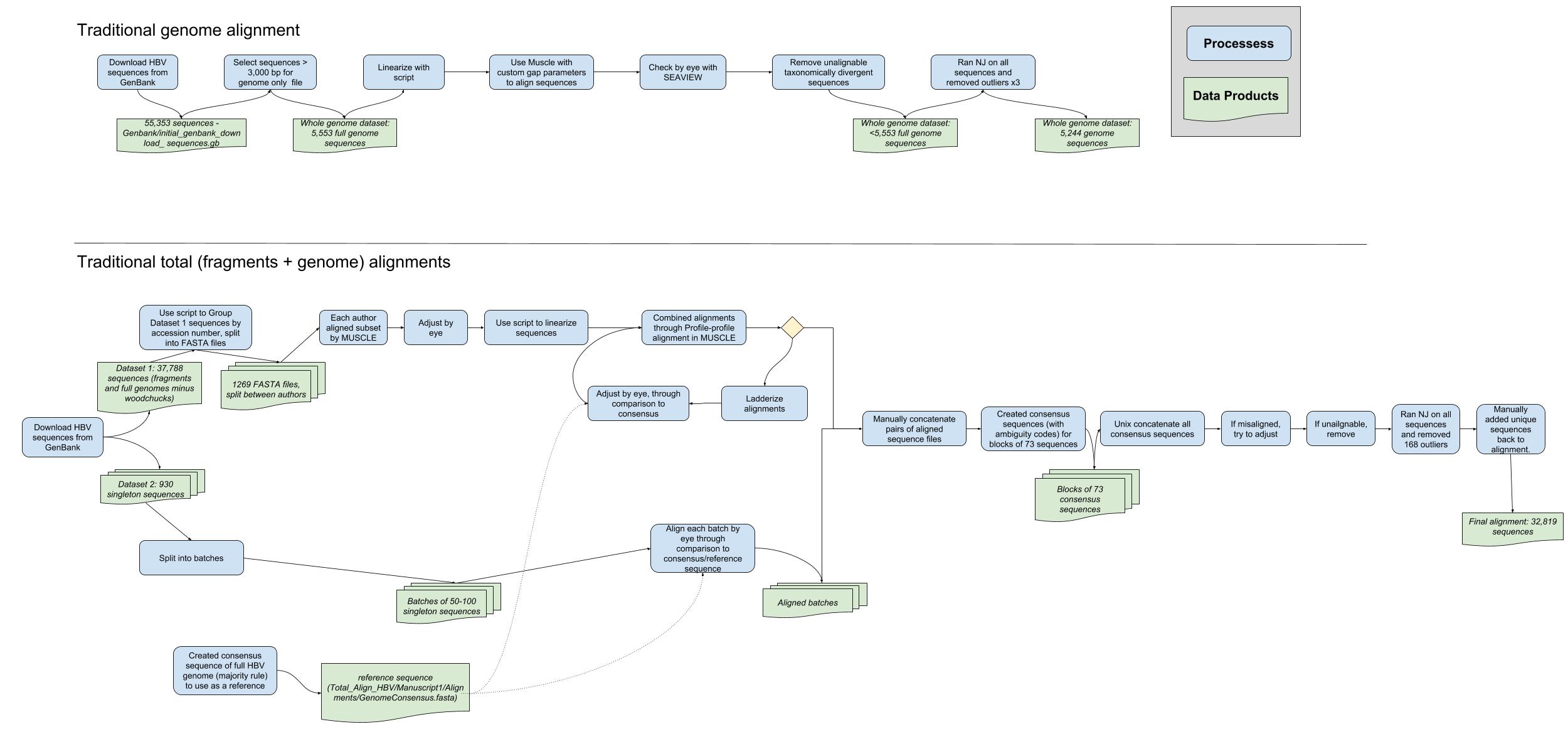

Supplement: Figure S1 — Workflow undertaken to align publicly available hepatitis B virus sequences using a traditional approach (automated alignment followed by manual adjustments). Genome alignment (top) and total (fragmentary + genomes; bottom) data sets are both illustrated. Blue boxes represent processes (i.e., specific analyses), and green shapes represent data products (e.g., files). [file peerj-07-6142-s001.jpg]

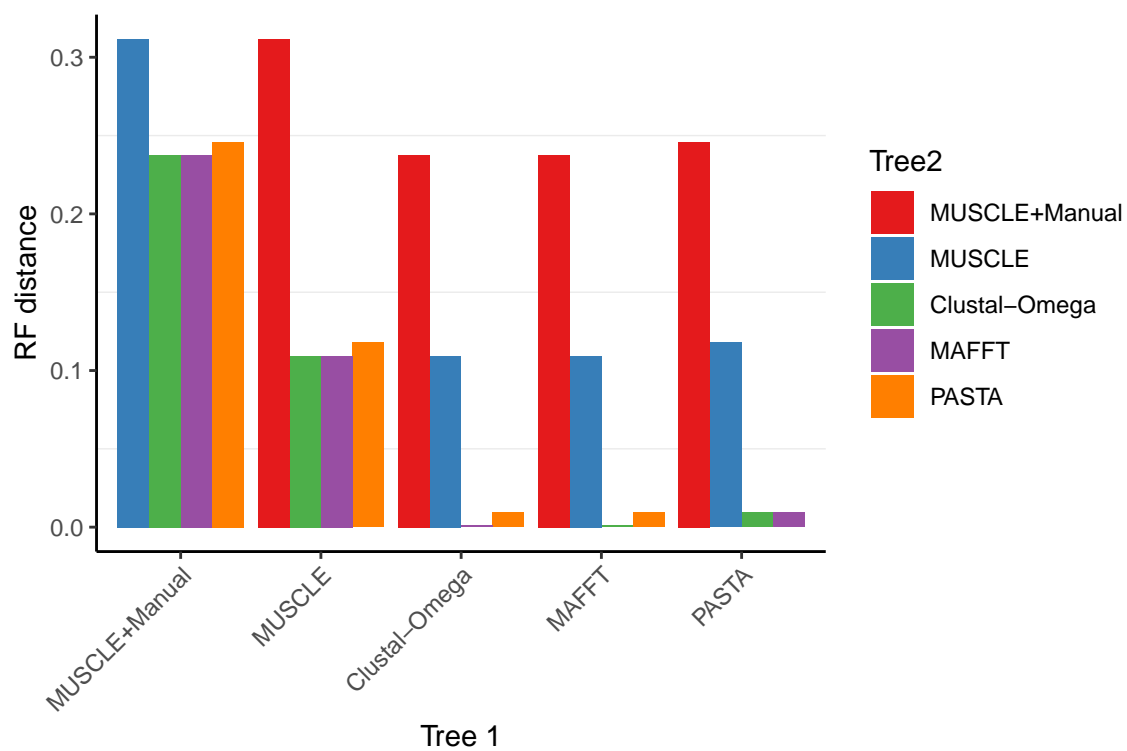

Supplement: Figure S2 — The results show that PASTA, Clustal Omega and MAFFT result in similar tree topology for highly supported edges. [file peerj-07-6142-s002.pdf]

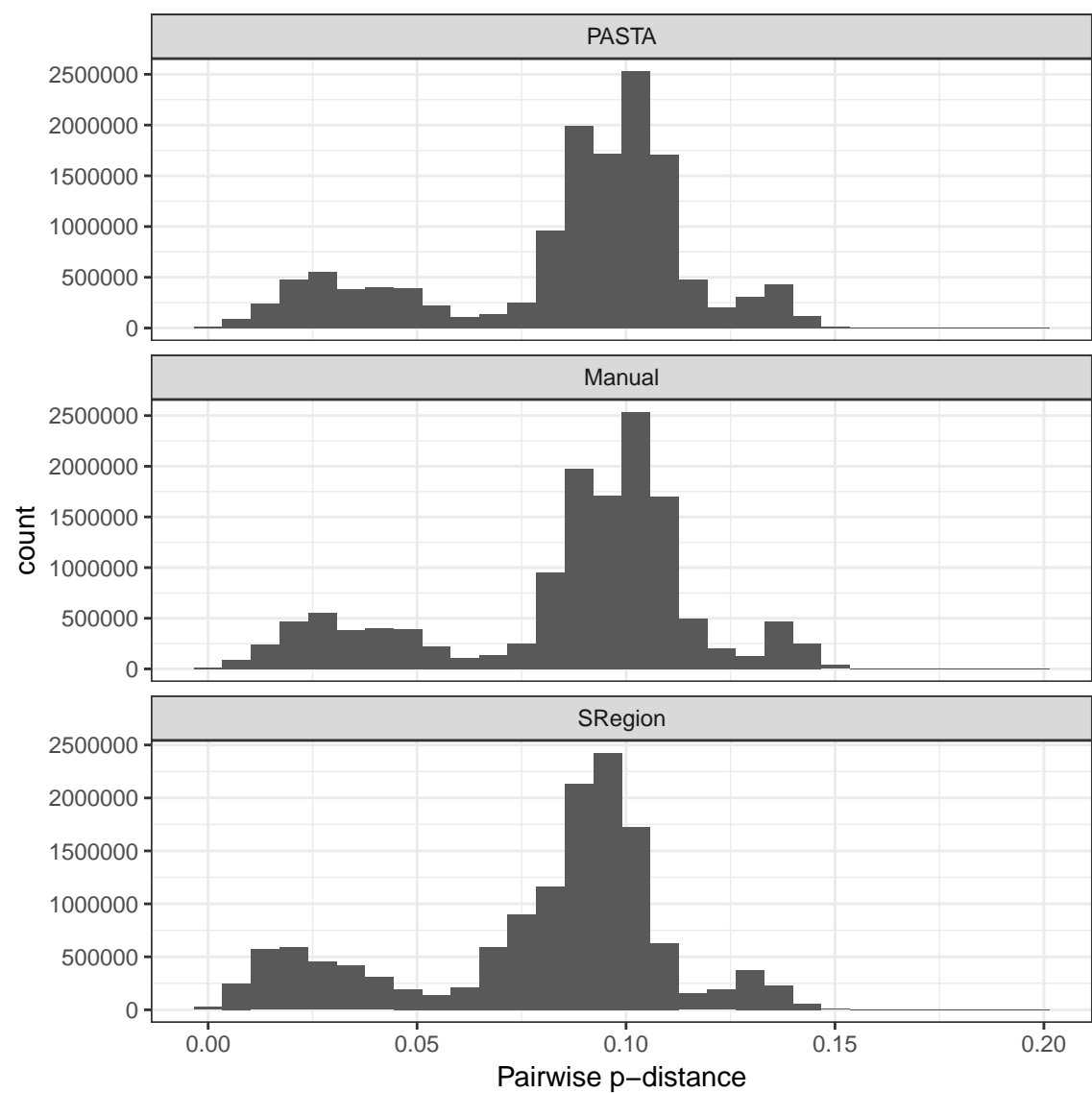

Supplement: Figure S3 — The alignments methods PASTA (top), Manual (middle), S-region (bottom) show similar pairwise p-distance distributions. [file peerj-07-6142-s003.pdf]
